# Slow yoga breathing improves mental load in working memory performance and cardiac activity among yoga practitioners

**DOI:** 10.3389/fpsyg.2022.968858

**Published:** 2022-09-14

**Authors:** Singh Deepeshwar, Rana Bal Budhi

**Affiliations:** Division of Yoga and Life Sciences, Cognitive Neuroscience Laboratory, Swami Vivekananda Yoga Anusandhana Samsthana (S-VYASA), Bangalore, KA, India

**Keywords:** pranayama, working memory, attention, heart rate variability (HRV), autonomic functions, yoga breathing

## Abstract

This study investigated the immediate effect of slow yoga breathing (SYB) at 6 breaths per minute (bpm) simultaneously on working memory performance and heart rate variability (HRV) in yoga practitioners. A total of 40 healthy male volunteers performed a working memory task, ‘n-back’, consisting of three levels of difficulty, 0-back, 1-back, and 2-back, separately, before and after three SYB sessions on different days. The SYB sessions included alternate nostril breathing (ANB), right nostril breathing (RNB), and breath awareness (BAW). Repeated measures analysis of variance showed a significant reduction in reaction time (ms) in 2-back condition immediately after ANB (−8%), RNB (−8%) and BAW (−5%) practices. Similarly, the accuracy was improved in the 0-back condition after RNB (4%), and in the 2-back condition after ANB (6%) and RNB (6%) practices. These results suggest that SYB practice enhances cognitive abilities (8–9%) related to memory load and improves the functioning of cardiac autonomic activity, which is required for the successful completion of mental tasks.Trial registered in the Clinical Trials Registry of India (CTRI/2018/01/011132).

## Introduction

Cognitive abilities affect everyday life and being able to perform mental tasks efficiently, is important for psychological well-being. These abilities include mental workload, attention, multitasking ability, reasoning, etc. (Tucker-Drob, 2009). Excessive stress affects autonomic and cardiovascular function, which is connected to impaired cognitive and mental functions (da Silva et al., 2009; Delliaux et al., 2019). However, for intended results in task performance, mental faculties and cognitive performance necessitate a certain amount of stress (Atkinson, 1957). Previous research suggested that elevated heart rate variability (HRV) mitigates the effect of mental stress on frontal lobe functions (McIntosh et al., 2021). The HRV is a promising index of cardiac autonomic activity that reflects oscillatory intervals between consecutive heartbeats. The beat-to-beat interactions (i.e., R-R interval) reflect the parasympathetic nervous system (PNS) and the sympathetic nervous system (SNS) input from sinoatrial nodes (Shaffer and Ginsberg, 2017). The dynamic interaction of R-R intervals has frequently been linked with various mind–body interventions that include yoga breathing practices also (Raghuraj and Telles, 2008; Pal et al., 2014; Bhagat et al., 2017). A recent study has shown that slow yoga breathing increases parasympathetic activity and baroreflex sensitivity in yoga practitioners compared to naïve (Anasuya et al., 2020).

Yoga breathing is a three-step method that manipulates nostril breathing: deep inhalation (*puraka*), complete exhalation (*rechaka*), and the cessation of breath (*khumbhaka*). Hatha yogic books have described several slow yoga breathing techniques (Muktibodhananda, 1998; Saraswati, 2012) and in the current study, we focused on the most convenient and popular type of yoga breathing techniques, i.e., (i) alternate nostril breathing (*Nadisodhana*; ANB), (ii) right nostril breathing (*Suryanuloma-viloma*; RNB), and (iii) breath awareness (BAW). The manipulations in yoga breathing allow alteration in autonomic activity and higher-order cognitive functions that are yet to be fully understood. The relationship between the R-R interval of autonomic measures following RNB and ANB has been explored in previous investigations (Raghuraj and Telles, 2008; Pal et al., 2014). The authors reported that RNB practice has sympathomimetic effects that were expressed by increased heart rate (HR), metabolism, and O_2_ consumption (Telles et al., 1994; Sarang and Telles, 2006), while the practice of ANB at a fixed rate of 5 breaths per min (BPM) showed higher low frequency (LF) power, LF/HF ratio, and a lower high frequency (HF) power of HRV measures (Sharma et al., 2014; Vinay et al., 2016). In contrast, Srivastava et al. reported that practice of 15 min of ANB for 8 weeks reduces HR, indicating a parasympathetic activity (Srivastava et al., 2005). Overall studies suggest that RNB practice increases SNS and ANB increases PNS activity (Lee and Ghiya, 2012; Bhavanani et al., 2014). However, there is a scarcity of research that has explored how slow yoga breathing techniques influence neurocognitive activities. A few research investigations suggest that yoga breathing affects autonomic activity, which may also alter brain functions that include attention and memory (Raghuraj et al., 1998; Santhanam Kumar et al., 2020). A previous investigation has revealed that the practice of ANB improves the performance in the target detection task (evaluated through the P300 oddball task) compared to the BAW session (Telles et al., 2013). According to the studies cited above, yoga breathing not only alters sympathetic or parasympathetic autonomic activity but also enhances mental performance. As far as we know there is no study that has explored the influence of slow yoga breathing (SYB) simultaneously on mental load during working memory and changes in autonomic indices of HRV. Hence, the present study aimed to evaluate the effect of slow yoga breathing practices (SYB) at a fixed rate of approx. 6 bpm simultaneously on: (1) working memory, (2) HRV in different working memory conditions, and (3) HRV during SYB sessions.

## Materials and Methods

### Participants

We recruited 40 young right-hand dominant males to participate in the present study, 27 undergraduate and 13 postgraduate students, from a yoga University, in South India (aged between 18 and 30 years, mean ± SD; 22.3 ± 4.2 years). The sample size was calculated with G-Power software (Erdfelder et al., 1996) by fixing the alpha at 0.05, power at 0.95, and an effect size of 0.71 based on the LF power in normalized unite (n.u.) mean and SD of the ANB session of an earlier study (Pal et al., 2014). The calculated sample size was 37, but due to possible dropout, we considered 40 participants in the study. The study was conducted between August 2016 and July 2018. Each participant was selected based on the following criteria, these were (i) Participants should have a minimum of 6 months of yoga practice, (ii) participants should not take any medication which influences cognitive functions, (iii) participants should not be involved in any other ongoing research activities, (iv) only male participants were enrolled in the study because cognitive, autonomic, and respiratory variables are known to change with the menstrual cycle (Ramírez et al., 2015; Anjana et al., 2017), and (v) participants who are willing to participate and adhere to the study protocol. The exclusion criteria were (i) addicted to a drug or on medicine, (ii) surgery in the last 6 months, and (iii) any type of known impairment.

The study was approved by the Institutional Ethics Committee (IEC) of the S-VYASA University, Bangalore, and a signed consent form was obtained from each participant. The trial was registered at Clinical Trial Registry—India (CTRI/2018/01/011132).

### Design of the study

Even though participants had prior experience of yoga breathing, the participants were given a 30-min orientation to practice SYB at 6 bpm for 1 month (5 days/week) under the guidance of an experienced yoga trainer before data acquisition in the laboratory. The purpose of the training was to maintain uniformity in SYB sessions during data acquisition in the laboratory. The SYB sessions included (i) right-nostril breathing (*suryanuloma viloma*; RNB), (ii) alternate nostril breathing (*nadishodhana*; ANB), and (iii) breath awareness (spontaneous breathing from both nostrils with awareness; BAW) on different days but at the same time for each participant. All eligible participants who completed the orientation program were given a practice session of a neurocognitive task.

**Figure 1:**
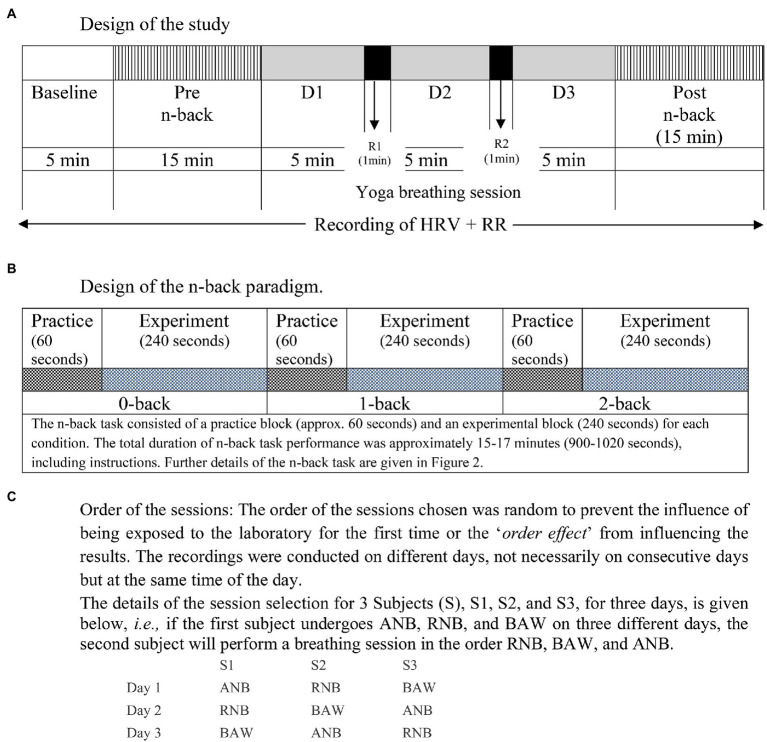
Schematic representation of the design of the study. **(A)** Design of the study, **(B)** Design of the n-back paradigm, **(C)** Order of the sessions.

Before and after each SYB session, participants were asked to perform a neurocognitive task, i.e., the visual n-back task consisted of three conditions (0-back, 1-back, and 2-back) as described in assessments. The electrocardiogram (ECG) and respiration were recorded during the baseline (BS), neurocognitive (Pre and Post n-back), and during SYB sessions. The schematic presentation of the study design and the neurocognitive task is given in Figure 1A and Figure 1B, respectively. The order of SYB sessions was random as presented in Figure 1C.

### Procedure

All assessments were done in the Cognitive Neuroscience laboratory of the S-VYASA University. The demographic information (age, education, physical activity, and socioeconomic status) and medical history were obtained before administering the working memory task. Before recordings, each participant was asked about the previous and current day activities such as physical exertion, diet, and quality of sleep because these activities are known to influence autonomic and cognitive functions. The recording was done at 5: 00–8: 30 AM and 4: 00–6: 30 PM. The assessments included the administration of a visual n-back task (0-back, 1-back, and 2-back) during which continuous ECG and respiration were recorded.

Participants were seated comfortably in a light dampening and sound attenuated chamber on a straight back armchair with elbows flexed at an angle of 90°. Each participant performed a visual-n back task before and after yoga breathing sessions.

#### Visual N-back task

Working memory (WM; Kirchner, 1958) was assessed using a computerized visual n-back task, which consisted of three conditions, i.e., 0-back, 1-back, and 2-back. Alphabet stimuli were presented in pseudorandom sequences in a fixed central location on a Dell computer using SuperLab™ 5.0 (Cedrus Corp, San Pedro, CA, United States). All participants used their right-hand index and middle fingers to press one of two buttons, denoting “target” and “nontarget” on a response pad. In the 0-back condition, the target was any letter that matched a pre-specified letter (i.e., “X”). Thus, this condition required sustained attention but no WM demand. In the 1-back condition, the target was any letter identical to the letter immediately preceding it (i.e., the letter presented one trial back). In the 2-back condition, the target was any letter that was identical to the letter presented two trials back. Each stimulus appeared on the screen for 500 ms, followed by a blank screen for another 500 ms in 0-back, 700 in 1-back, and 1,500 in 2-back for responses. Further, for preparation, 1,000 ms was given for the interstimulus interval (ISI) between each alphabet. Participants completed one block of trials with each of the three conditions and each block consisted of 150 trials. A short break (5–20 s) between blocks was provided to allow participants to rest. Before the actual task, participants were given practice blocks (of 50 trials each) before each condition with feedback on their performance until they demonstrated that they understood the task and their performance stabilized. Reaction times (RTs) and error scores were obtained for each trial.

#### Autonomic changes using HRV

**Figure 2:**
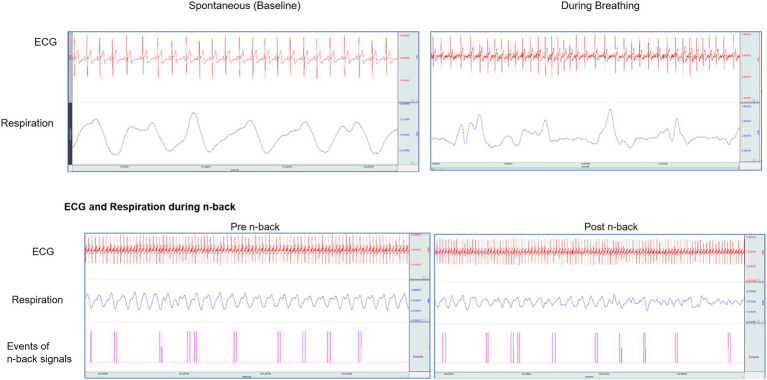
Original traces of ECG, respiration in one participant while breathing at their baseline, during slow yoga breathing, and in n-back task performance.

The ECG and respiration were recorded throughout a session, which lasted for 55–60 min (before, during n-back, and breathing epochs were used for analysis) using a 16-channel polygraph system (MP 100 BIOPAC, Acknowledge software, BIOPAC System Inc., United States). The ECG was recorded using Ag/AgCl pre-gelled electrodes (Tyco Healthcare, Germany), and recordings were made with Standard Limb Lead I configuration. Data were acquired at the sampling rate of 1,024 Hz and were analyzed offline to obtain the HRV, which gives an indication of the balance in the autonomic innervation to the heart. The HRV can be modified by complex factors, including respiration. Noise-free data were included for analysis. A sample of original traces of ECG, and respiration during spontaneous breathing at baseline, during yoga breathing, and during the n-back performance is presented in Figure 2.

#### Respiration rate

Respiration was concurrently monitored in an attempt to remove the contribution of respiration to the HRV, especially lower frequencies (around 0.1 Hz) as the interaction between the HRV and respiration is traditionally known. The respiratory rate was recorded using a volumetric pressure transducer fixed around the trunk about 8 cm below the lower costal margin as the participants sat erect.

### Slow yoga breathing (SYB) sessions

There were three SYB sessions given to each participant as described below.

*Right nostril yoga breathing* (RNB) or *Suryanuloma viloma pranayama* practice involves breathing exclusively inhalation and exhalation through the right nostril while the left nostril is gently occluded. Whereas, *alternate nostril yoga breathing* (ANB) practice involves breathing through left and right nostrils alternately. In this practice, the thumb and ring finger of the right hand were used to manipulate or occlude the nostril through the gesture known as *nasika mudra*. This helps to manipulate breath through nostrils with ease during *pranayama*.

During the active control BAW session, participants focused their awareness on the breath. Each participant was asked to observe the flow of air passing through nasal passages with closed eyes.

### Data extraction

The simultaneous HRV and respiration were recorded during 4 states, i.e., (i) baseline (5 min), (ii) pre-n-back (12–15 min), (iii) SYB session (15 min), and (iv) post-n-back (12–15 min). Before analysis, the acquired data were visually reviewed offline, and only noise-free data were considered for further analysis. The original traces of ECG and respiration during the entire sitting of one participant are represented in Figure 2. The Fast Fourier Transform (FFT) was used to obtain the HRV power spectrum. The following HRV variables were studied in frequency domain measures: (i) low frequency (LF) band (0.05–0.15 Hz), (ii) high frequency (HF) band (0.15–1.50 Hz), and (iii) the LF/HF ratio. The low frequency and high-frequency band values were expressed as normalized units. The following components of time-domain HRV were analyzed: (i) mean RR interval (the mean of the intervals between the instantaneous heart rate), (ii) RMSSD (square root of the squared difference of successive NN intervals; where NN = normal to normal intervals), (iii) NN50 (the number of successive NN intervals with differences greater than 50 ms), and (iv) pNN50 (proportion derived by dividing NN50 by the total number of NN intervals; where NN50 is the number of interval differences of successive NN intervals greater than 50 ms).

### Data analysis

The raw data of continuous variables were inspected visually with histograms and tested with the Kolmogorov–Smirnov test. Statistical analysis was done using Statistical Package for Social Science (SPSS) version 21. The effect of SYB sessions on reaction time and accuracy of the n-back task was analyzed with repeated measures of ANOVA with two Within-subject factors: Factor 1-SYB sessions (ANB vs. RNB vs. BAW), and Factor 2-time (pre n-back-*vs* post n-back). Further, SYB sessions’ effect on different conditions of the n-back task was analyzed with a repeated-measures ANOVA. A similar analysis was done for HRV during SYB sessions with a repeated-measures ANOVA with two Within-subject factors: Factor 1-SYB sessions (ANB, RNB, and BAW) and Factor 2-State (Baseline, During 1, During 2, and During 3 (D1, D2, D3)) for each parameter.

### Results

There were no dropouts in the study. The group mean values ± S.D. reported for (1) the reaction time and accuracy of the n-back task, (2) HRV during n-back, and (3) HRV during SYB sessions are given in Tables 1,2,3, respectively.

**Table 1:** Effect of yoga breathing on reaction time (ms) and accuracy (%) of n-back task.

Session	0-Back			1-Back			2-Back		
	Pre	Post	% change	Pre	Post	% change	Pre	Post	% change
**Reaction time (in ms)**									
ANB	500.23 ± 55.76	503.42 ± 66.36	0.64	528.41 ± 61.18	522.86 ± 84.95	−1.05	708.15 ± 169.23	647.32 ± 147.39 **	−8.59
RNB	503.74 ± 74.70	494.14 ± 55.24	−1.90	548.72 ± 117.82	535.15 ± 91.75	−2.47	745.44 ± 164.47	683.76 ± 159.04*	−8.27
BAW	509.01 ± 62.87	498.83 ± 71.03	−2.00	551.91 ± 99.77	547.17 ± 114.80	−0.86	694.06 ± 136.50	652.58 ± 148.12**	−5.98
**Accuracy (in %)**									
ANB	86.48 ± 12.58	88.32 ± 9.37	2.13	89.36 ± 10.60	90.12 ± 8.68	0.85	78.26 ± 10.54	83.44 ± 8.97***	6.62
RNB	86.23 ± 9.89	90.10 ± 7.09**	4.49	89.02 ± 9.88	92.03 ± 5.95	3.38	74.63 ± 9.93	79.49 ± 10.81**	6.50
BAW	86.95 ± 10.60	88.74 ± 9.80	2.06	85.35 ± 13.93	87.86 ± 9.84	2.95	71.78 ± 15.08	74.80 ± 12.26	4.21

*p < 0.05, **p < 0.01, ***p < 0.001 (two-tailed test) = Pre/Post of 0-, 1-, 2-back tasks; %change was calculated using the formula = (Post/Pre) × 100–100.

Values are group mean ± SD (*n* = 40).

**Table 2:** HRV measures during an n-back performance before and after yoga breathing. Values are group mean ± SD (n = 40).

Session	Variables	0-Back		1-Back		2-Back	
		Pre	Post	Pre	Post	Pre	Post
**Frequency domain measures**							
ANB	LF(nu)	53.93 ± 22.20	61.17 ± 19.74**	56.21 ± 17.09	62.36 ± 17.43*	58.92 ± 21.82	63.20 ± 17.10*
	HF(nu)	45.95 ± 22.11	38.73 ± 19.68**	43.65 ± 17.05	37.48 ± 17.37*	41.00 ± 21.82	36.66 ± 17.09*
	LF-HF ratio	1.81 ± 1.59	2.73 ± 3.02*	1.76 ± 1.49	2.34 ± 1.81	2.33 ± 1.99	2.53 ± 2.40
RNB	LF(nu)	52.25 ± 19.23	57.81 ± 15.82*	56.87 ± 17.66	62.28 ± 15.58	58.57 ± 14.38	64.69 ± 16.50
	HF(nu)	47.60 ± 19.12	42.00 ± 15.75*	43.02 ± 17.63	37.56 ± 15.56	41.30 ± 14.36	35.16 ± 16.49
	LF-HF ratio	1.87 ± 3.02	1.98 ± 2.35	1.80 ± 1.35	2.14 ± 1.42	1.72 ± 0.99	2.60 ± 1.84**
BAW	LF(nu)	53.44 ± 17.65	56.60 ± 20.72	56.38 ± 18.91	57.47 ± 18.27	57.00 ± 19.52	59.58 ± 12.96
	HF(nu)	46.40 ± 17.63	43.31 ± 20.70	43.46 ± 18.89	42.29 ± 18.37	42.82 ± 19.58	40.27 ± 13.06
	LF-HF ratio	1.57 ± 1.46	2.02 ± 1.92	2.00 ± 2.12	1.91 ± 1.51	1.77 ± 1.10	1.76 ± 1.08
**Time domain measures**							
ANB	Mean HR	71.45 ± 8.71	73.00 ± 8.93	74.91 ± 10.10	74.99 ± 9.44	75.40 ± 10.07	75.60 ± 9.12
	Mean RR	853.10 ± 109.09	839.71 ± 116.04	820.79 ± 117.02	817.61 ± 113.62	813.55 ± 113.67	810.43 ± 105.06
	NN50	80.23 ± 51.78	70.08 ± 50.78	75.08 ± 56.68	67.41 ± 44.08	66.13 ± 58.52	67.87 ± 47.93
	pNN50	29.80 ± 20.392	25.95 ± 19.442*	26.63 ± 20.315	24.09 ± 17.656	21.26 ± 18.682	22.83 ± 17.821
	RMSSD	55.44 ± 27.84	50.92 ± 26.55 *	52.93 ± 28.93	50.30 ± 27.11	46.40 ± 23.45	46.98 ± 24.15
RNB	Mean HR	71.08 ± 8.91	72.10 ± 8.50	73.34 ± 8.66	73.50 ± 8.45	74.44 ± 8.44	74.69 ± 7.80
	Mean RR	862.54 ± 108.81	848.41 ± 104.30	834.25 ± 103.59	833.00 ± 99.45	820.97 ± 97.94	816.85 ± 92.17
	NN50	85.26 ± 50.20	66.13 ± 38.28	78.26 ± 47.46	69.54 ± 42.44	67.90 ± 45.64	62.21 ± 42.91
	pNN50	31.31 ± 18.862	24.34 ± 14.830**	26.88 ± 16.155	24.92 ± 16.282	21.94 ± 14.959	20.58 ± 14.656
	RMSSD	60.60 ± 31.04	51.26 ± 25.30 **	56.32 ± 23.61	52.25 ± 28.14	48.06 ± 20.30	43.40 ± 18.74
BAW	Mean HR	72.50 ± 8.94	72.04 ± 7.83	73.18 ± 8.95	73.89 ± 7.76	74.43 ± 8.67	74.97 ± 7.71
	Mean RR	844.48 ± 99.74	848.84 ± 91.06	835.25 ± 101.27	824.97 ± 86.40	821.60 ± 98.19	813.18 ± 82.99
	NN50	86.00 ± 53.33	73.69 ± 51.12*	79.77 ± 59.02	71.28 ± 49.30*	70.46 ± 55.77	65.33 ± 50.53**
	pNN50	31.45 ± 19.850	27.75 ± 20.575	28.37 ± 21.502	25.05 ± 17.931	23.17 ± 19.034	21.41 ± 16.740
	RMSSD	60.41 ± 35.40	60.17 ± 42.01	55.77 ± 37.16	53.73 ± 28.18	52.13 ± 29.17	49.58 ± 26.98

*p < 0.05, **p < 0.01, ***p < 0.001 (two-tailed test) = BS/D1, BS/D2, BS/D3.

**Table 3:** HRV measures at baseline and during yoga breathing practices. Values are group mean ± SD (*n* = 40).

Session	Variables	During SYB			
		BS	D1	D2	D3
**Frequency domain measures**					
ANB	LF(nu)	58.19 ± 22.13	86.17 ± 15.73***	88.29 ± 13.39***	84.71 ± 13.45***
	HF(nu)	41.65 ± 22.18	13.79 ± 15.68***	11.64 ± 13.21***	15.26 ± 13.41***
	LF-HF ratio	2.81 ± 4.76	15.62 ± 14.05***	14.24 ± 9.47***	10.43 ± 7.61***
RNB	LF(nu)	59.61 ± 20.48	81.51 ± 21.29***	78.75 ± 20.96***	77.81 ± 20.56**
	HF(nu)	40.27 ± 20.50	18.43 ± 21.25***	21.20 ± 20.89***	22.14 ± 20.56**
	LF-HF ratio	2.82 ± 4.55	12.67 ± 11.69***	10.57 ± 11.70**	10.24 ± 11.29**
BAW	LF(nu)	52.38 ± 16.67	63.10 ± 20.65***	65.51 ± 21.03***	66.24 ± 20.04***
	HF(nu)	47.55 ± 16.63	36.84 ± 20.63***	34.41 ± 21.00***	33.71 ± 20.07***
	LF-HF ratio	2.13 ± 5.30	4.48 ± 7.75**	4.06 ± 5.77*	4.61 ± 6.64**
**Time domain measures**					
ANB	Mean HR	71.83 ± 9.14	77.21 ± 9.02***	76.60 ± 8.42***	75.59 ± 7.99***
	Mean RR	855.80 ± 113.91	799.17 ± 99.38***	803.38 ± 93.86***	813.18 ± 90.23***
	NN50	85.08 ± 53.33	86.13 ± 43.16	84.15 ± 46.41	84.97 ± 48.54
	pNN50	85.08 ± 53.33	86.13 ± 43.16	84.15 ± 46.41	84.97 ± 48.54
	RMSSD	51.51 ± 26.22	60.55 ± 33.81	54.29 ± 27.10	60.27 ± 31.72
RNB	Mean HR	70.39 ± 8.46	76.22 ± 8.02***	76.26 ± 6.65**	75.98 ± 7.00***
	Mean RR	875.82 ± 106.90	807.38 ± 85.19***	804.46 ± 75.09***	807.57 ± 79.43***
	NN50	92.97 ± 55.10	92.03 ± 40.90	84.41 ± 44.45	84.77 ± 46.94
	pNN50	29.83 ± 18.48	24.97 ± 11.9	22.16 ± 10.78	23.12 ± 14.01
	RMSSD	54.87 ± 27.73	61.13 ± 27.57	67.43 ± 46.56	64.28 ± 36.82
BAW	Mean HR	71.27 ± 9.79	72.01 ± 8.54	72.00 ± 8.06	71.62 ± 8.45
	Mean RR	862.66 ± 115.53	850.86 ± 94.47	851.83 ± 93.95	856.12 ± 97.41
	NN50	88.90 ± 62.62	88.05 ± 56.15	89.64 ± 53.53	89.49 ± 54.37
	pNN50	27.7 ± 22.23	27.15 ± 19.33	27.9 ± 20.06	28.9 ± 20.52
	RMSSD	56.90 ± 38.00	55.05 ± 29.10	57.46 ± 31.85	56.78 ± 32.16

*p < 0.05, **p < 0.01, ***p < 0.001 (two-tailed test) = BS/D1, BS/D2, BS/D3

Variable and initials used: HYB, Hatha yoga breathing; ANB, Alternate nostril breathing; RNB, Right nostril breathing; BAW, Breath awareness; BS, Baseline; D1-D3, During three epochs 5 min, each yoga breathing; LF, Low frequency; HF, High frequency; RMSSD, Root mean square of the successive differences; NN50, the number of pairs of successive NNs that differ by more than 50 ms; pNN50, the proportion of NN50 divided by the total number of NNs; SDNN, the standard deviation of NN intervals; TINN, triangular interpolation of NN interval.

#### N-back task

The ANOVA results for reaction time showed a significant main effect on Time [*F*_(1,39)_ = 12, *p* < 0.001, *ηp*^2^ = 0.36]. Similarly, in accuracy, there was a significant main effect on session [*F*_(2,78)_ = 6.99, *p* < 0.01, *ηp*^2^ = 0.15] and time [*F*_(1,39)_ = 22.14, *p* < 0.001, *ηp*^2^ = 0.24].

*Post-hoc* test with Bonferroni adjustment was performed for each condition of n-back. The results showed a significant decrease in the reaction time of 2-back after ANB (*p* < 0.01), RNB (*p* < 0.05), and BAW (*p* < 0.01) practices. However, participants showed a better reaction time after ANB compared to other practices. Participants showed a significant improvement in accuracy of the 2-back condition (*p* < 0.01) after ANB practice, and significant improvement in 0-back (*p* < 0.01) and 2-back (*p* < 0.01) conditions after RNB practice, while there was no change in accuracy of the n-back performance after BAW practice when compared to pre-scores.

#### HRV during n-back

The ANOVA results for measures of frequency domain analysis showed a significant main effect on time in LF [*F*_(1,37)_ = 17.39, *p* < 0.001, *ηp*^2^ = 0.32], HF [*F*_(1,37)_ = 17.51, *p* < 0.001, *ηp*^2^ = 0.32], and LF-HF ratio [*F*_(1,38)_ = 9.46, *p* < 0.01, *ηp*^2^ = 0.20]. Similarly, in measures of time-domain analysis, there was a significant main effect on time in RMSSD [*F*_(1,37)_ = 7.9, *p* < 0.01, *ηp*^2^ = 0.18], NN50 [*F*_(1,37)_ = 9.58, *p* < 0.01, *ηp*^2^ = 0.21], and pNN50 [*F*_(1,37)_ = 7.5, *p* < 0.01, *ηp*^2^ = 0.17].

*Post-hoc analysis with Bonferroni correction* showed that after ANB practice there was a significant increase in the LF power during 0-back (*p* < 0.01), 1-back (*p* < 0.05), and 2-back (*p* < 0.05), and in the LF/HF ratio during 0-back (*p* < 0.05). And there was a decrease in the HF power during 0-back (*p* < 0.01), 1-back (*p* < 0.05) and 2-back (*p* < 0.05). Results on post RNB practice showed that there was a significant increase in LF power during 0-back (*p* < 0.05) and LF/HF ratio during 2-back (*p* < 0.01) and a decrease in the HF power in 0-back (*p* < 0.05).

ANB practice revealed a significant decrease in pNN50 and RMSSD (*p* < 0.05) in time-domain HRV measurements during the post-0-back condition compared to the pre-condition. There was a significant decrease in pNN50 (*p* < 0.01), and RMSSD (*p* < 0.01) when compared to pre 0-back condition following the RNB session. After BAW practice, there was a significant decrease in NN50 during post 0-back (*p* < 0.05), post 1-back (*p* < 0.05) and post 2-back (*p* < 0.01) when compared to respective pre-conditions.

#### HRV during SYB sessions

The ANOVA results of HRV measures of frequency domain analysis during SYB showed significant main effect on sessions in LF [*F*_(2, 76)_ = 25.63, *p* < 0.001, *ηp*^2^ = 0.40], HF [*F*_(2,76)_ = 27.7, *p* < 0.001, *ηp*^2^ = 0.41], and LF-HF ratio [*F*_(2,76)_ = 19.46, *p* < 0.001, *ηp*^2^ = 0.34]. Likewise, main effect on during in LF [*F*_(1.68, 63.80)_ = 52.30, *p* < 0.001, *ηp*^2^ = 0.58], HF [*F*_(1.68, 63.80)_ = 51.92, *p* < 0.001, *ηp*^2^ = 0.58], and LF-HF ratio [*F*_(2.44, 92.59)_ = 26.17, *p* < 0.001, *ηp*^2^ = 0.42].

Also, there was a significant interaction effect on sessions × during in LF [*F*_(3.73, 141.61)_ = 4.37, *p* < 0.01, *ηp*^2^ = 0.10], HF[*F*_(3.73, 141.65)_ = 4.32, *p* < 0.01, *ηp*^2^ = 0.10], LF-HF ratio [*F*_(5.41, 201.68)_ = 6.34, *p* < 0.001, *ηp*^2^ = 0.14]. Similarly, HRV measures of time domain analysis during SYB showed a significant main effect on sessions in Mean HR [*F*_(2, 76)_ = 7.05, *p* < 0.05, *ηp*^2^ = 0.16] and Mean RR [*F*_(1, 74)_ = 6.08, *p* < 0.01, *ηp*^2^ = 0.14]; and interaction effect between session × during in Mean HR [*F*_(5.30, 196.16)_ = 10.34, *p* < 0.001, *ηp*^2^ = 0.22], Mean RR [*F*_(4.28, 158.4)_ = 6.55, *p* < 0.001, *ηp*^2^ = 0.21], and RMSSD [*F*_(4.5, 166.5)_ = 4.58, *p* < 0.001, *ηp*^2^ = 0.11].

*Post-hoc analysis with Bonferroni correction* in frequency domain analysis showed that there was a significant increase in LF power, LF/HF ratio, and decrease in HF power during ANB, RNB, and BAW sessions (*p* < 0.05; *p* < 0.01; *p* < 0.001). Similarly, time-domain measures also showed a significant increase in Mean HR, and Mean RR, during ANB and RNB sessions (*p* < 0.05; *p* < 0.01; *p* < 0.001). However, there were no changes in time-domain measures of the BAW session.

**Figure 3:**
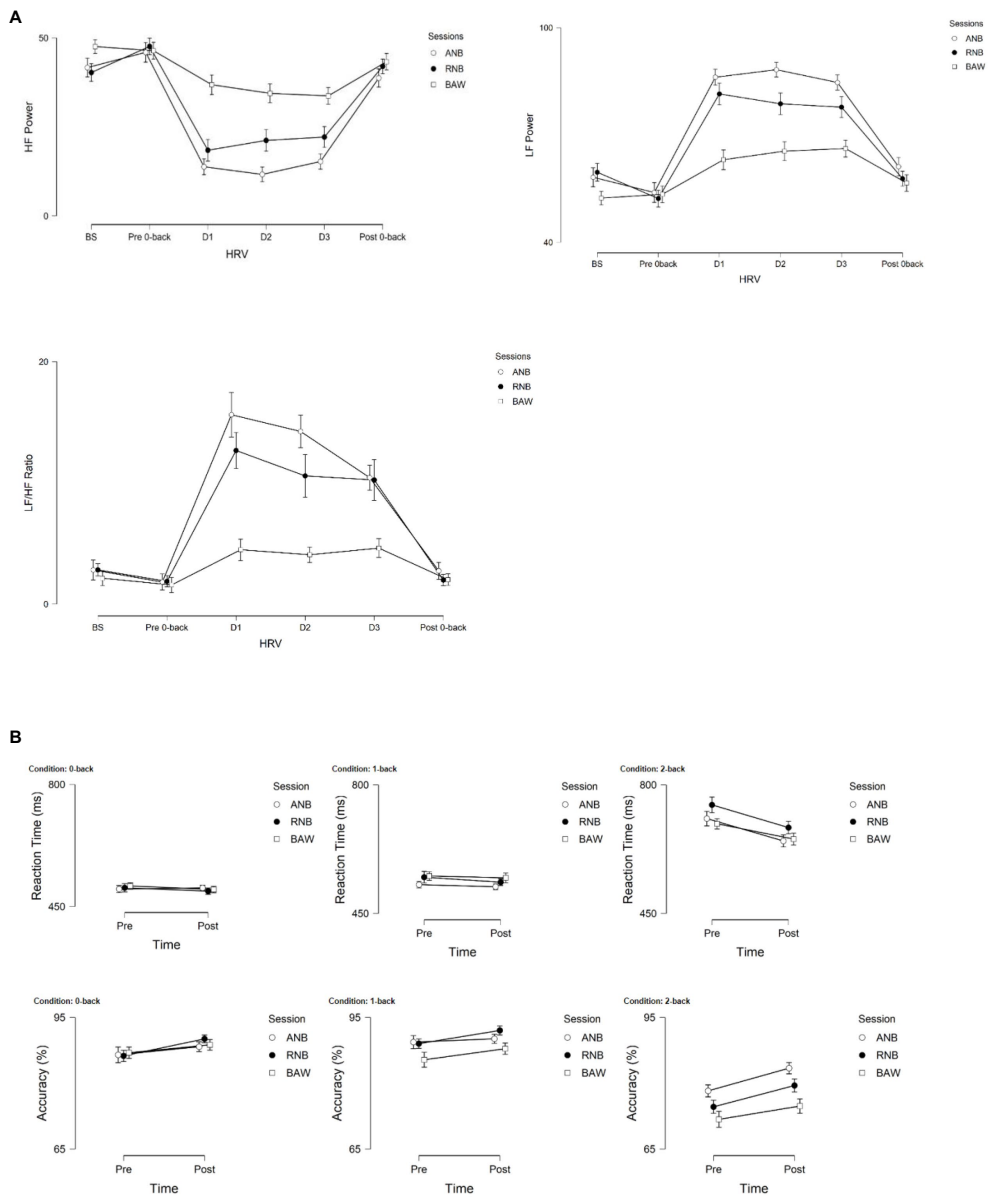
**(A)** Graphical representations of LF power, HF power, and LF/HF ratio during baseline, n-back (pre-post), and during SYB (*n* = 40). **(B)** Reaction time (ms) and accuracy (%) of n-back conditions (0-, 1-, and 2-back) following SYB.

The graphical presentation of LF power, HF power, and LF/HF ratio during baseline, n-back, and yoga breathing are given in Figure 3A. Similarly, the changes in reaction time and accuracy of n-back conditions before and after SYB sessions are given in Figure 3B.

The breathing rate did not show any significant change during the session, and is therefore not reported in the results.

## Discussion

To our knowledge, this is the first study to investigate the immediate effect of SYB practice simultaneously on mental workload with working memory tasks and changes in the measures of HRV. The study results revealed that SYB at 6 bpm significantly increases SNS activity and improves mental processes during working memory performance. Our findings indicate that practicing yoga breathing can increase the physiological load, i.e., linked to the significant increase in SNS activity. SNS activity was moderately elevated during the mental load in the present study which is required to complete working memory tasks efficiently (Knight et al., 2020). This has been reflected in the results by faster reaction time (ms) in working memory performance with better accuracy, suggesting that slow yoga breathing through one nostril may improve mental functioning in young adults (Giuliano et al., 2018). Therefore, it can be suggested that the SYB practice may enhance processing speed and the ability to discriminate between target and non-target events in the complex n-back task, particularly in the 2-back condition.

According to previous research, cognitive tasks require significant levels of sympathetic activity or parasympathetic inhibition to complete the task (Berntson et al., 1996; Melis and Van Boxtel, 2001; Melis and van Boxtel, 2007). Parasympathetic withdrawal is associated with “feed-forward” input from higher brain centers to the medullary cardiovascular center that triggers a rapid increase in heart rate which is mediated by temporarily reduced cardiac parasympathetic neural activity. However, sympathetic activation is triggered with moderate to maximal intensity of practice and mediated by the systemic sympathoadrenal activation (Billman, 2013; White and Raven, 2014). During working memory performance, HRV results indicate parasympathetic withdrawal after ANB and RNB breathing practice. That may be linked to improving performance on a task that needs more mental effort. In addition to this, after BAW there was a decrease in reaction time for the 2-back task without any statistically significant changes in accuracy.

An earlier study demonstrated that participants practicing yoga breathing at a rate of 6 bpm showed sympathetic arousal which might be due to an increase in physiological load during yogic breathing (Raghuraj and Telles, 2008). Overall, the findings showed that slow breathing considerably raises the LF power and LF/HF ratio, reflecting the predominance of sympathetic activity and sympathovagal balance, respectively. Slow yoga breathing directly influences vagal tone or baroreflex sensitivity that modulate the sinoatrial node, thereby increasing HR oscillation and HRV (Bernardi et al., 2001). The ANB and RNB showed a temporary withdrawal of parasympathetic activity and a concomitant increase in HR that can be linked to higher baroreflex sensitivity and oxygen saturation (Mason et al., 2013). Further, slow breathing studies have demonstrated beneficial effects on cardiorespiratory functions in patients with cardiac surgery, prehypertension, asthma, and major depressive disorder (Karavidas et al., 2007; Lin et al., 2012). The findings of the present study are consistent with the previous study (Lin et al., 2014) which implies that slow yoga breathing practices may be beneficial for the physical and mental health of clinically healthy individuals, mainly due to improvements in the subjective feeling of relaxation. However, the mechanism of action of yoga breathing on autonomic and cognition is not fully understood.

In the neurovisceral integration model, greater parasympathetic activity is linked to selective attention, whereas heightened sympathetic activity is associated with an efficient attention-related processing (Giuliano et al., 2018). In line with the aforementioned findings, we observed that cognitive task performance was improved after slow and controlled SYB (ANB and RNB) sessions, and even after a simple BAW session. The SYB practice may activate specific brain regions such as the dorsal pons, periaqueductal grey matter, cerebellum, hypothalamus, thalamus, lateral and anterior insular cortices in the human brain as revealed in the volitional controlled slow breathing study (Nagai et al., 2015). A recent study reported that yoga breathing and meditation increase synchronization between respiratory and attentional systems *via* LC activity thereby improving attentional, emotional, and physiological functioning (Melnychuk et al., 2018). Moreover, unilateral yoga breathing at a slow rate (5–8 bpm) demonstrated increased oxygenation and blood volume in the left prefrontal cortex (PFC) after the RNB session as compared to the BAW session (Singh et al., 2015). Though speculation, the above-mentioned brain areas may be associated with the enhancement of autonomic functions during yoga breathing and while performing a neurocognitive task. There might be several other pathways involved in yoga breathing practice that directly or indirectly influence autonomic activity and cognitive functions that needs to be explored in future studies.

## Limitations

A number of limiting factors are likely to account for the current study: the sample size was relatively small, also the lack of a control group (those never exposed to yoga practice), and study outcomes are limited to male yoga practitioners only. Further, during the neurocognitive task, the remnant effect of the practice block could not be ruled out due to technical limitations. The results cannot be extrapolated to generalize the neurocognitive performance as a whole because they are restricted to the immediate impact of yoga breathing practice on neurocognitive function. Moreover, the autonomic changes during pre n-back task might have a carryover effect on HRV in breathing sessions, which should be controlled in a further study. Furthermore, the study findings can only be generalized to healthy individuals and future studies could concentrate on participants with depression or people with mental health issues. Future research should extend the present methodology to a wider array of experimental tasks and examine simultaneous assessments of HRV, and brain imaging techniques (fMRI, fNIRS, or EEG) related to the long-term practice of meditation and yoga breathing. However, within our study, SYB sessions increased parasympathetic withdrawal which was reflected in the improvement of working memory performance when compared with spontaneous breathing in the BAW session.

## Conclusion

The present study empirically showed cardiac autonomic arousal during SYB among yoga practitioners. Additionally, moderate SNS activation may support physiological arousal, which is necessary for efficient mental functioning and working memory performance. The outcome of the study suggests that yoga breathing may have the potential to enhance coping abilities by optimizing heightened physiological arousal and facilitating attentional processes. Therefore, SYB may be useful for bringing positive changes in the autonomic activity related to clinical conditions (dysautonomia, attention deficit hyperactive disorder [ADHD], and major depressive disorder [MDD]), which should be investigated in future studies. Furthermore, SYB immediate practice prior to a mental task may facilitate better responses to autonomic activity that is associated with neurocognitive performance.

## Data availability statement

The raw data supporting the conclusions of this article will be made available by the authors, without undue reservation.

## Ethics statement

The studies involving human participants were reviewed and approved by Institutional Ethics Committee of SVYASA - RES/IEC-SVYASA/009/2015. The patients/participants provided their written informed consent to participate in this study. Written informed consent was obtained from the individual(s) for the publication of any potentially identifiable images or data included in this article.

## Author contributions

SD: conceptualization, funding acquisition, methodology, project administration, formal analysis, data curation, validation, interpretation of the data, and writing—original drafting of the work. RB: acquisition, resources, software for analysis, supervision, and validation. All authors contributed to the article and approved the submitted version.

## Funding

This study was funded by the Science and Engineering Research Board (SERB) [YSS/2015/000734], Department of Science and Technology (DST), New Delhi, Government of India.

## Conflict of interest

The authors declare that the research was conducted in the absence of any commercial or financial relationships that could be construed as a potential conflict of interest.

## Publisher’s note

All claims expressed in this article are solely those of the authors and do not necessarily represent those of their affiliated organizations, or those of the publisher, the editors and the reviewers. Any product that may be evaluated in this article, or claim that may be made by its manufacturer, is not guaranteed or endorsed by the publisher.
